# Data on how students׳ involvement with ENACTUS can affect their decision for entrepreneurship

**DOI:** 10.1016/j.dib.2018.08.047

**Published:** 2018-08-23

**Authors:** Stephen Oluwatobi, Damilare Oshokoya, Aderemi Atayero, Olumuyiwa Oludayo

**Affiliations:** Hebron Startup Lab, Covenant University, Nigeria

## Abstract

The data are descriptions of the responses of the students that attended the ENACTUS (ENtrepreneurial ACTion US) Nigeria leadership conference 2018, which held from February 19 to 21, 2018. Hence, this data article describes the age range of the students, the amount of ENACTUS projects they have been involved in, their willingness to start their ventures, when they would like to launch their ventures and the industries they would like to venture into. The Google Doc Online Form was used to design the questionnaire used for sourcing the data. 109 respondents, of 333 that attended the conference, completed the questionnaire.

**Specifications Table**

**Table:** 

Subject area	*Economics and Business*
More specific subject area	*Entrepreneurship and Innovation*
Type of data	*Graphs, Charts, Figure*
How data was acquired	*An online questionnaire, using Google Doc Form, was used to gather the data*[3].
Data format	*Raw*
Experimental factors	*The samples consist of students from higher institutions in Nigeria*
Experimental features	*Students’ involvement in enterprise activities affect their decision to become entrepreneurs.*
Data source location	*Higher Institutions in Nigeria*
Data accessibility	*Data is available with this article*

**Value of the data**

• The data will be useful for analyzing students’ willingness to become entrepreneurs, start their ventures, when they will like to start the ventures and what industry they are interested in starting their ventures in.
• It is possible to use the data to measure and determine the kind of activities higher education institutions need to establish to inspire, encourage and enable students’ desires to become entrepreneurs.
• The data will help tertiary institution see the impact of Enactus in building the overall entrepreneurial-mindedness required of their students.
• The data will help also clarify the impact of entrepreneurial student organizations on building students’ entrepreneurial interest.
• This will enable further research on entrepreneurial environment and model for tertiary institution.

## 1 Data

**Fig. 1:**
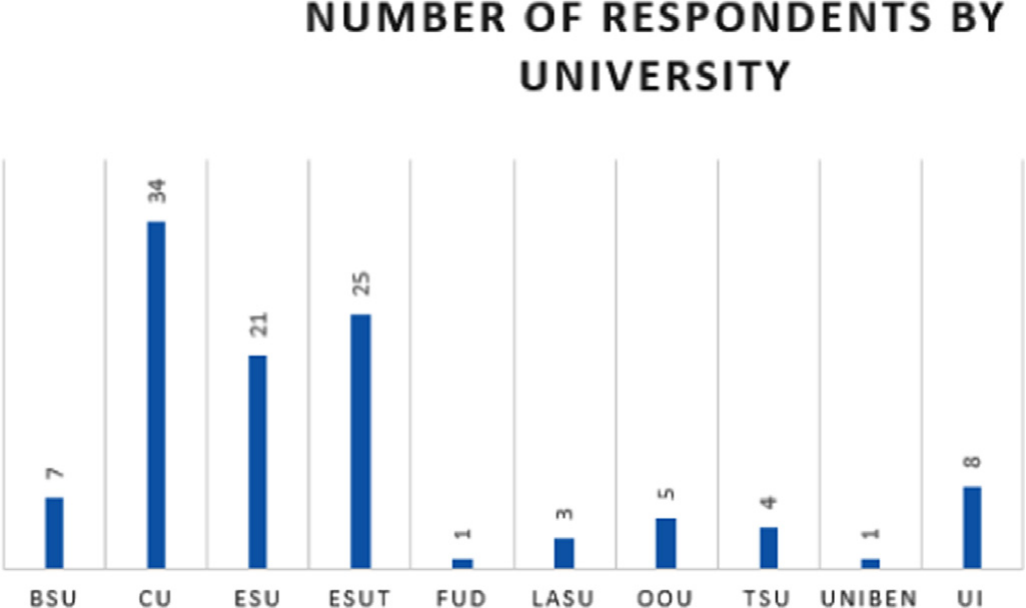
Number of respondents by university.

**Fig. 2:**
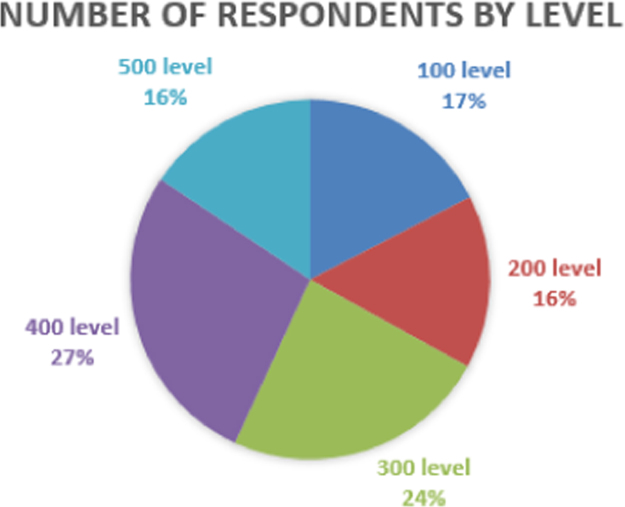
Number of respondents by level.

**Fig. 3:**
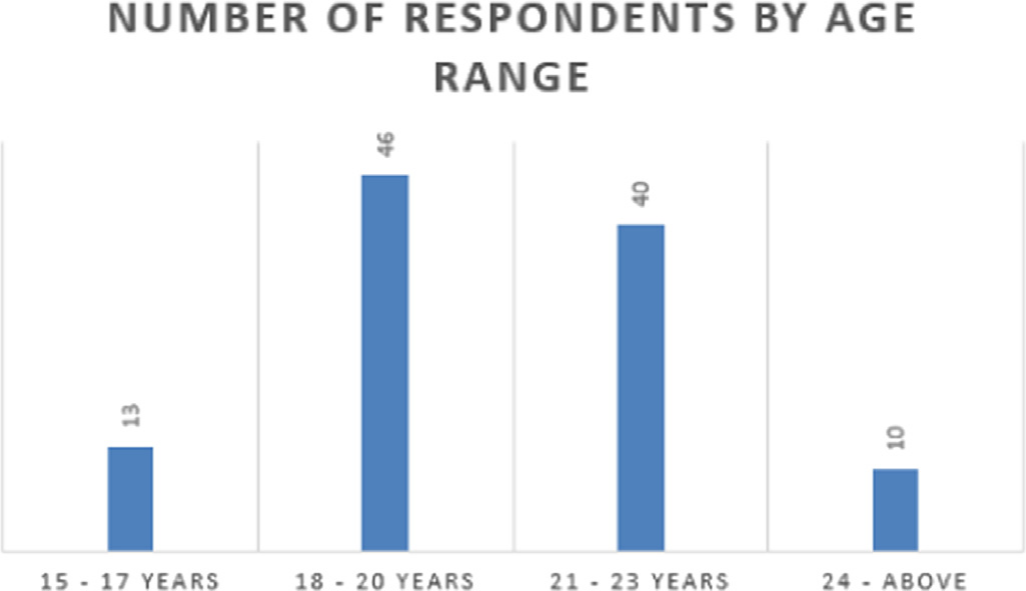
Number of respondents by age range.

**Fig. 4:**
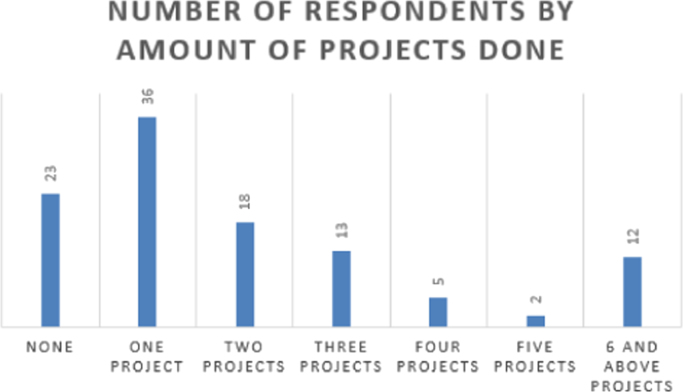
The number of projects the students have been involved in.

**Fig. 5:**
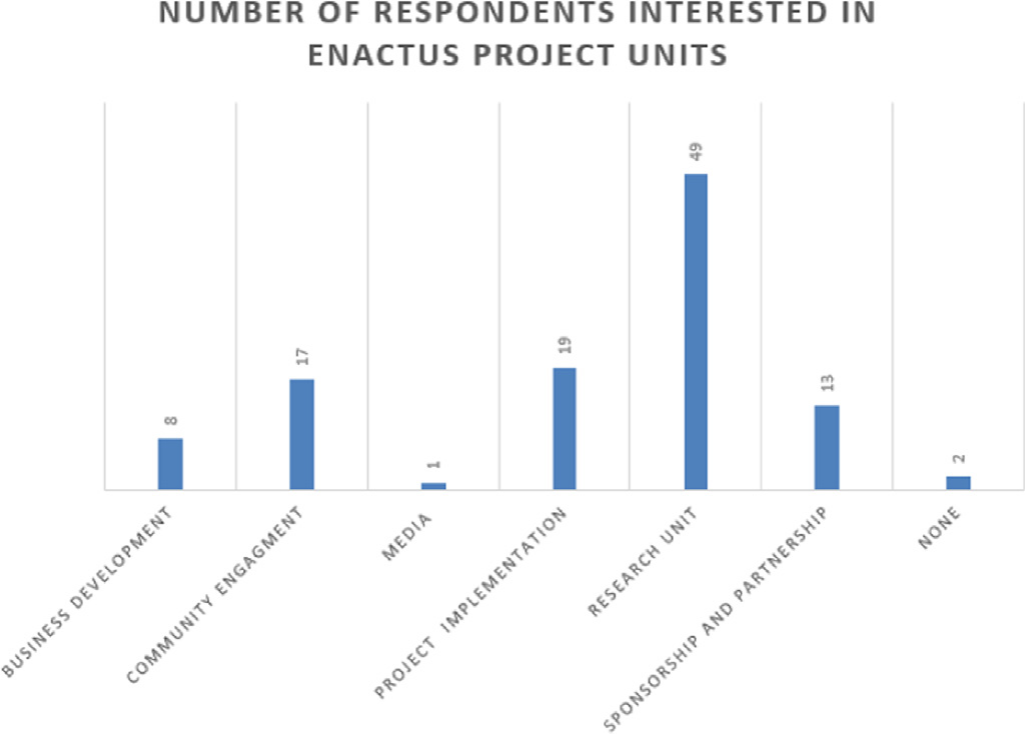
Number of students interested in ENACTUS project units.

**Fig. 6:**
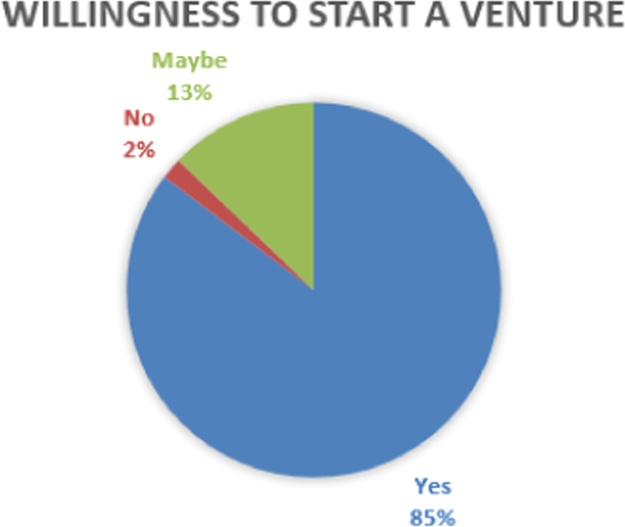
Willingness to start a venture.

**Fig. 7:**
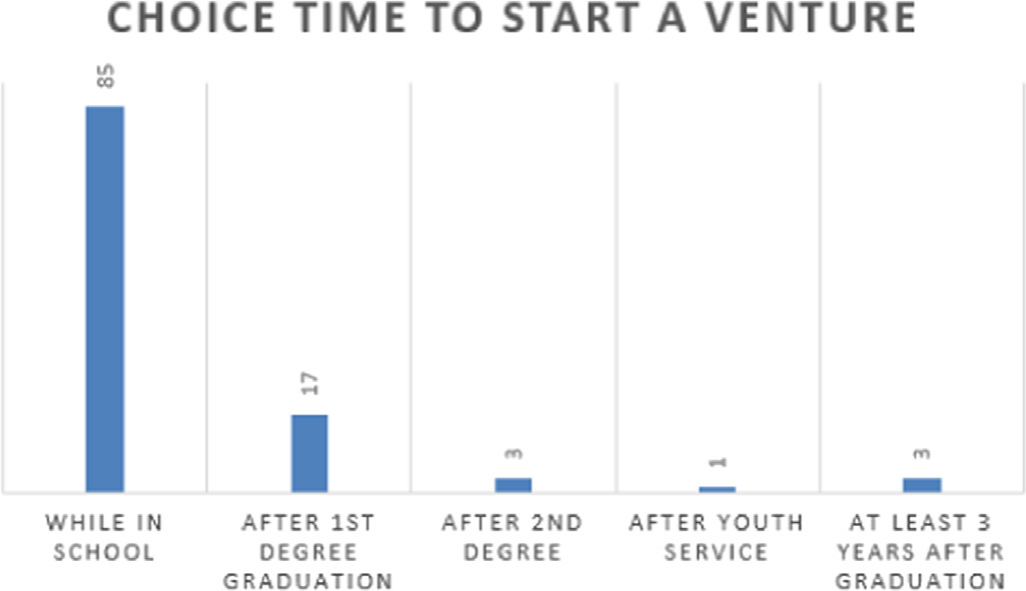
When students desire to start their ventures as a result of their Involvement in ENACTUS.

**Fig. 8:**
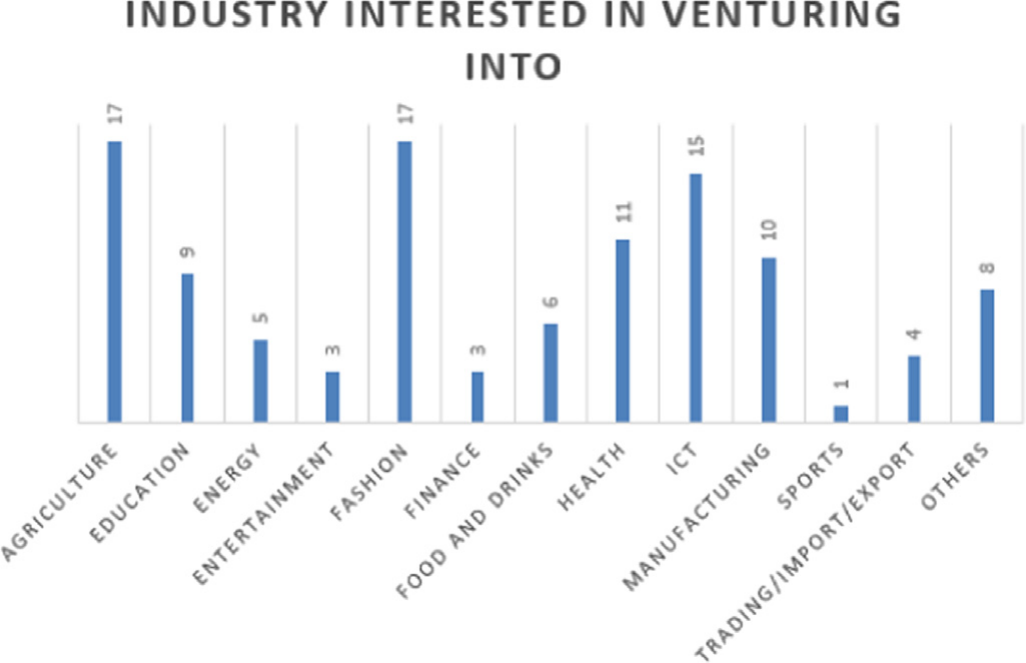
The industries students are interested in venturing into.

The data are presented in Fig. 1, Fig. 2, Fig. 3, Fig. 4, Fig. 5, Fig. 6, Fig. 7, Fig. 8. They include the number of respondents by University, the number of respondents by level, the number of respondents by age range, the number of projects the students have been involved in, the number of students interested in each of the ENACTUS *(ENtrepreneurial ACTion US)* project units [2], the respondents’ willingness to start a venture, when the respondents desire to start their venture, and the industry the respondents desire to venture into respectively.

Students’ involvement in ENACTUS requires that they be involved in entrepreneurially-related projects that challenge them to think entrepreneurially [2]; hence, Fig. 4 shows the number of projects the respondents have been involved in. Besides this, ENACTUS student teams are expected to function in any of six units in order to ensure properly balanced teams [1]. These six units include business development, community engagement, media, project implementation, research unit, and sponsorship and partnership. Fig. 5 shows the respondents’ interests in these units. As part of exploring the impact of ENACTUS activities on the students, respondents were also questioned on how their involvement affects their decision to start their own ventures. The data on this are presented in Fig. 6. In addition, respondents were also asked when they would like to start their venture. Their responses are as presented in Fig. 7. Furthermore, the respondents where queried as to the sector or industry they would like to venture into. The data showing their responses are shown in Fig. 8.

## 2 Experimental design, materials and methods

The Google Doc Online Form was used to design the questionnaire used for sourcing the data. 109 respondents, of 333 that attended the conference, completed the questionnaire. Their responses are described in Fig. 1, Fig. 2, Fig. 3, Fig. 4, Fig. 5, Fig. 6, Fig. 7, Fig. 8. The link to the questionnaire [3] was made available to the students that attended the ENACTUS Leadership Conference 2018 that held on February 19th to 21st, 2018 at Covenant University, Ogun state, Nigeria. The Conference is an annual leadership retreat for ENACTUS student leaders and executives to be trained and equipped on project management, community development, teamwork, leadership and business. The responses from the respondents are presented in the following figures:

The respondents came from 10 higher institutions in Nigeria as shown in Fig. 1.

Level in Fig. 2 refers to the year of the respondent in school; hence, 100 level, 200 level, 300 level, 400 level and 500 level refers to 1st year, 2nd year, 3rd year, 4th year and 5th year respectively.
